# The Influence of Shape Changing Behaviors from 4D Printing through Material Extrusion Print Patterns and Infill Densities

**DOI:** 10.3390/ma13173754

**Published:** 2020-08-25

**Authors:** Seokwoo Nam, Eujin Pei

**Affiliations:** Department of Design, Brunel University, London UB8 3PH, UK; Eujin.Pei@brunel.ac.uk

**Keywords:** 4D printing, material extrusion, shape changing behavior, shape memory polymers, print pattern, infill density, polylactic acid

## Abstract

Four-dimensional printing (4DP) is an approach of using Shape Memory Materials (SMMs) with additive manufacturing (AM) processes to produce printed parts that can deform over a determined amount of time. This research examines how Polylactic Acid (PLA), as a Shape Memory Polymer (SMP), can be programmed by manipulating the build parameters of material extrusion. In this research, a water bath experiment was used to show the results of the shape-recovery of bending and shape-recovery speed of the printed parts, according to the influence of the print pattern, infill density and recovery temperature (Tr). In terms of the influence of the print pattern, the ‘Quarter-cubic’ pattern with a 100% infill density showed the best recovery result; and the ‘Line’ pattern with a 20% infill density showed the worst recovery result. The ‘Cubic-subdivision’ pattern with a 20% infill density demonstrated the shortest recovery time; and the ‘Concentric’ pattern with a 100% infill density demonstrated the longest recovery time. The results also showed that a high temperature and high infill density provided better recovery, and a low temperature and low infill density resulted in poor recovery.

## 1. Introduction

According to Pei et al [1], the development of four-dimensional printing (4DP) has been encouraged by the rapid development of Additive Manufacturing (AM) and Shape Memory Materials (SMMs). Notably, 4DP parts can morph or change shape, depending on the passage of time and the given environment conditions [2]. They have a potential to enable complex actuation devices and moving components to be built, in which sensors, mechanical parts and batteries can be eliminated. Smart materials, including Shape Memory Alloys (SMAs) or Shape Memory Polymers (SMPs) and other organic materials such as paper and wood, that are stimuli-responsive bio-composites, can be adapted for 4DP [3]. These SMMs can be restored to their original shape by environmental stimulation without external force after the programming step [4]. For shape recovery and deformation in 4DP, a stimuli is required to facilitate the shape change. Examples of stimuli include water, heat, pH, UV light, magnets, or combined sources of stimuli. Combining AM with smart materials through 4D printing, offers opportunities to design and build smart and active structures [5,6,7,8]. Statistics indicate that the market size for SMPs is expected to grow from US$1bn in 2021 to US$3.4bn in 2025 [9]. With the development of widely available SMPs, new 4DP applications within the product design industry are expected to grow. This paper focuses on SMPs, as polymeric materials are lighter, cheaper and easier to manufacture than SMAs, and they have a better recovery strain when compared to SMAs [10,11]. Felton et al. [12], demonstrated the use of AM with SMPs to produce self-assembly and self-folding parts. However, current research has not extensively examined how material extrusion build parameters may influence the shape memory effect of 4DP parts.

The aim of this study is to present a framework that will guide users to effectively predict the change in shape memory effect of bending, according to the influence of the build parameters using Polylactic acid (PLA). PLA has been chosen for this work due to its commercial availability and cost effectiveness. The outcome of this research will provide a better understanding of how shape changing behaviors can be effectively controlled through material extrusion print patterns and infill densities. Raviv et al. [13], found that intentionally specified build parameters can have a positive effect on the quality of the printed components and their mechanical properties. Therefore, research into the determination of optimal parameters is an important factor when designing 4DP parts [14]. A review of the literature has found that very little guidance is available for the selection of SMPs and whether build settings could influence the shape changing behavior.

## 2. Experimental Work

The first experiment consists of a material selection protocol to compare 16 different types of commercially available PLA materials, and to shortlist suitable PLA filaments. The second experiment is to produce printed samples based on the selection, to identify the best results. The third experiment is to investigate the use of 12 print patterns with different infill densities to ascertain the results of the shape memory effect (SME). The final experiment is to validate the results of the print patterns using a combination of different infill densities. The experiments were conducted with a consistent approach, by using the same AM hardware, software, and the same water bath set up.

### 2.1. Process for Material Selection and Shape Recovery Grades

**Figure 1:**
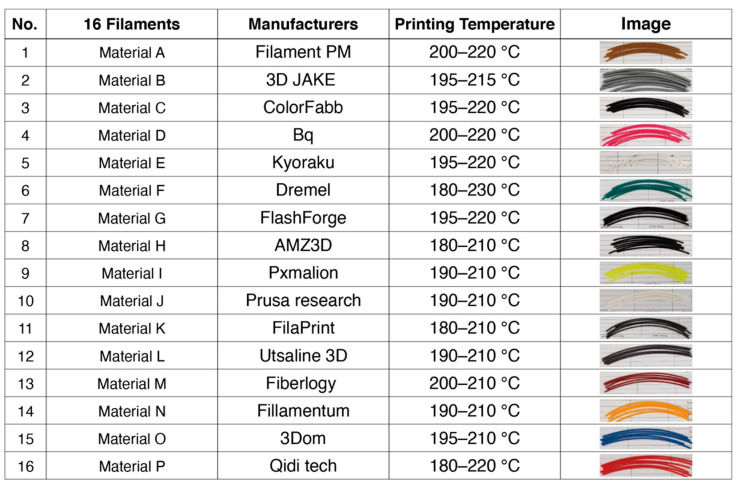
Sixteen different types of polylactic acid (PLA) samples.

Overall, 16 different types of PLA samples were purchased from different material suppliers as shown in Figure 1. Notably, 1.75 mm filaments were used, and they had a melting point for printing between 180 °C to 210 °C. Each reel of material was cut into strips 80 mm long. To record and grade the recovery effect, a grading system of 1 represented excellent recovery, returning to its full original shape; and a grade of 9 refers to no recovery. This is an important step to quantify the SME. A flat shape with a grade of 1 therefore indicates the best achievable recovery quality.

**Figure 2:**
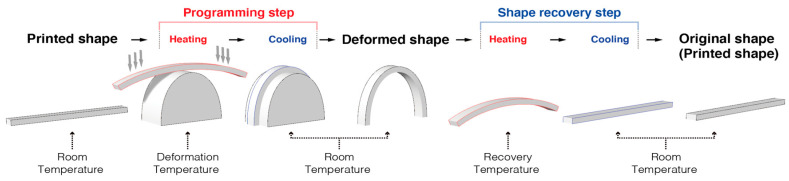
The processes involved in the shape change effect.

The SME process comprises three steps, encompassing the original shape, a temporary shape and the recovered shape [15]. A visual summary of the SME process is shown in Figure 2, which explains the systematic steps of programming, heating, cooling and recovery. The SME of the material is also determined by many factors, such as the material composition, the applied shape changing behavior, deformation rate, deformation temperature and recovery temperature [16].

For this experiment, the procedures described in [17,18] were referenced. For the water bath experiment, hot water was used to trigger the filaments and the AM parts. Programming of the raw PLA filaments first begins with a change in temperature of the water. An immersion circulator was used to heat the water in the water-bath to the deformation temperature. The 16 types of PLA filaments (Figure 2), each 80 mm long, were placed in 80 °C of water in a water-bath for 60 s, and they were manually deformed to the bending shape by using a deformation device. The fixation of the deformed PLA filaments was achieved by allowing them to naturally cool for 60 s at room temperature (25 °C). Next, the water-bath temperature was changed and set to the recovery temperature. The recovery temperature for the filament test was performed with four samples, with four different levels of recovery temperatures of 60 °C, 65 °C, 70 °C and 75 °C. This meant that there were four recovery temperature samples for each material, and a total of 16 types of PLA materials were applied. The deformed PLA filaments were placed into the recovery temperature of the water-bath, and the shape recovery result was recorded using a high resolution video camera mounted on a tripod. Effort was also made to ensure that the tests conducted were consistent for all samples being used.

### 2.2. Results of Shape Recovery with Shortlisted Materials

**Figure 3:**
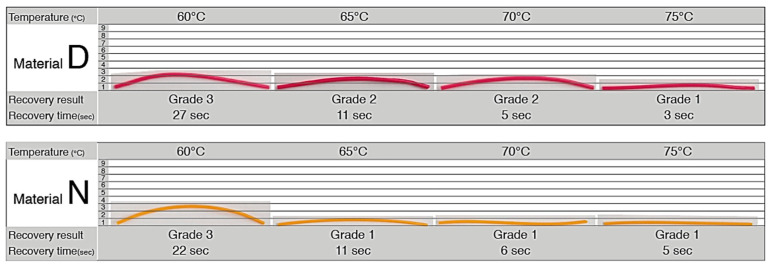
Material D and Material N showed the best recovery to their original shapes.

The results of the first stage described in Section 2.1 were systematically documented and placing the results of the PLA filaments onto a grading card. The grading card shows a grade of 1 that indicates the best achievable recovery quality, and a grade of 9 that indicates the worst performing recovery result. From the 16 shortlisted materials, Material D and Material N showed the best results, since a grading system of 1 represents excellent recovery to its original shape (Figure 3). In addition, the experiment enabled us to record the time it took to recover to its original shape. Figure 3 shows the recovery of four samples from Material D, and the recovery of four samples from Material N.

**Figure 4:**
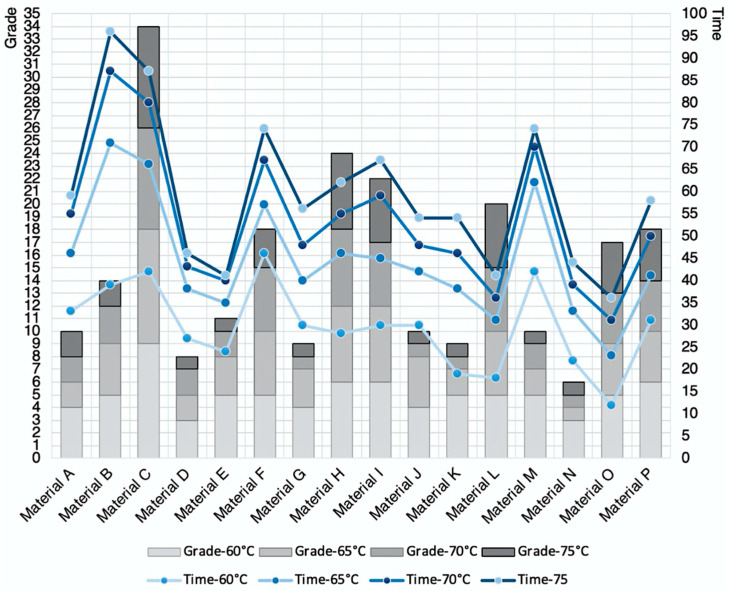
Recovery results of Materials A to P.

Next, the results of the recovery quality and the recovery time were compiled and quantitatively compared in a form of a histogram. To determine the best overall recovery result based on the 4 recorded temperatures, the total grade for the 4 samples of each material was calculated. For example, the calculation of Material N whereby 3 + 1 + 1 + 1 gave a total score of 6, which was the lowest score and the best recovery. It also showed that a better recovery result was achieved between 65 °C to 75 °C. A line graph was also plotted in Figure 4 to determine the recovery time, whereby the sum from the 16 filaments of each different temperatures was calculated. It was also found that generally, a higher temperature in the water bath produced a much faster reaction. Material O achieved the fastest recovery at 36 s. Material L and E achieved the second fastest recovery level at 41 s, and Material N achieved the third fastest recovery level at 44 s to return to its original shape. A decision was made to shortlist Material D and Material N, based on the performance achieved, and the availability and cost of the PLA filaments.

### 2.3. Additively Manufactured Samples

**Figure 5:**
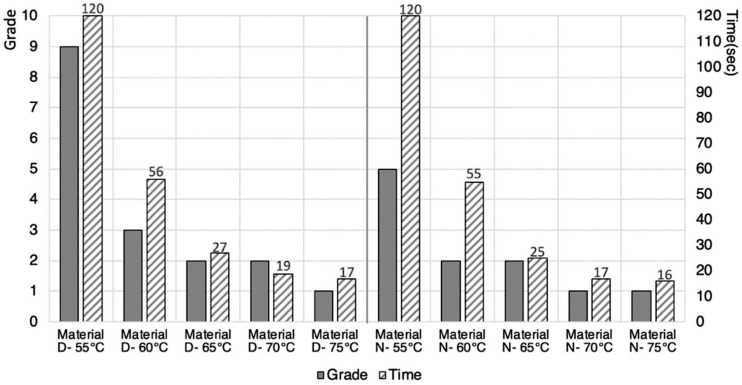
Histogram comparing the recovery grade and recovery time.

For the second step, standard rectangular samples measuring 80 mm × 6 mm × 3 mm were produced using filaments D and N selected as suitable PLA materials from the first step. The Qidi X-Pro Material Extrusion Printer was used, in which a consistent build setting of print temperatures of 210 °C, print speed at 50 mm/s, retraction time at 30 mm/min, retraction distance at 1.5 mm and infill density of 100% were used. The PLA samples were programmed for 60 s using deformation temperature of 80 °C and bent using the same deformation device. The fixation of the deformed PLA samples was allowed to cool for 60 s at room temperature (25 °C) after fixing to the deformation device. After PLA samples D and N were completely cooled down and programmed, the water-bath temperature was changed to the recovery temperature. To obtain more accurate results, the recovery temperature for the filament test was performed five times, by setting five different levels of recovery temperatures (55 °C, 60 °C, 65 °C, 70 °C and 75 °C). The deformed PLA samples were placed in the water-bath, and the progress of the shape recovery was recorded using the same video camera. The results in Figure 5 showed that almost the same results were obtained for both materials, for temperatures between 60 °C to 75 °C. When compared in detail, Material N showed better recovery quality. In terms of time, Materials D and N showed a slight difference, and both materials showed faster recovery time as the temperature increased. Overall, Material N showed a better recovery quality and a faster recovery than Material D. From this experiment, we can conclude that Material N would be used for the next step, focusing on aspects of print pattern and infill density.

### 2.4. Print Patterns and Infill Densities

**Figure 6:**
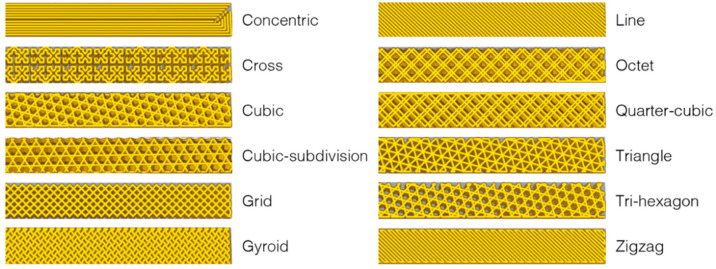
The 12 different types of print patterns available with Qidi Print.

The Qidi X-Pro Material Extrusion Printer was supplied with a Qidi Print slicer software, although other third-party programs such as Cura and Simplify3D are also compatible. There were total of 12 pre-defined patterns, including concentric, cross, cubic, cubic-subdivision, grid, gyroid, line, octet, quarter-cubic, triangle, tri-hexagon and zigzag patterns, as shown in Figure 6. The use of these patterns can affect the strength and flexibility of the printed parts. According to Zolfagharian et al [19], the bending angle of the 4DP actuators can be significantly affected by the type of pattern and number of layers. Therefore, the AM build parameters such as print patterns and the layer height could potentially influence the SME.

**Figure 7:**
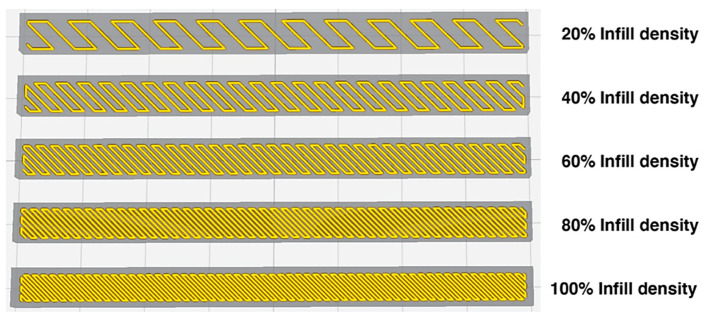
Example of infill densities from 20% to 100%.

In terms of infill density, this refers to the filling up of the space within the printed object or the density. The infill density is measured by means of a percentage. An object with a 100% infill volume represents a 100% object density. Objects with a higher percentage of the infill process consume more material, produce harder objects, and consequently take longer to produce. In contrast, objects with a lower percentage of the infill pattern consume less material, are lighter, and can be produced much faster [20]. A 0% infill density generally means that the object is empty, and 100% infill density means that the inside of the printed object is completely filled. Infill density can also affect the print strength, flexibility and the amount of material used. Figure 7 provides a visual example of the percentages of infill densities.

For this experiment, 12 patterns and 5 infill densities would be used for Material N, encompassing 20%, 40%, 60%, 80% and 100%. The same procedure performed in the experiments described in 2.1 and 2.3 was carried out. The printed sample measuring 80 mm × 6 mm × 3 mm would be produced. The experimental process through programming, cooling and recovery would be used, although it was decided to utilize only recovery temperatures of 65 °C, 70 °C and 75 °C to accelerate the study. This is because temperatures of 55 °C and 60 °C were found to be insufficient to produce a thermo-mechanical response from 4DP parts.

## 3. Results and Development of a Tool

Firstly, the experiments showed that a 20% infill density produced the best recovery result of the tri-hexagon pattern, with a grade of 2 at each 65 °C, 70 °C and 75 °C; and the worst result was the line pattern, with a grade of 9 at each degree. In terms of time, the cubic-subdivision pattern took the shortest duration of time of 6 s at 75 °C; and the concentric pattern took the longest duration of 21 s at 65 °C. Secondly, the experiments showed that a 40% infill density produced the best recovery result of the quarter-cubic, tri-hexagon and zigzag pattern, with a grade of 2 at 75 °C; and worst result from the line pattern, with a grade of 8 at 65 °C. In terms of time, the cross, gyroid and line pattern took the shortest duration of 10 s at 75 °C; and the concentric pattern took the longest duration of 25 s at 65 °C. Thirdly, the experiments showed that a 60% infill density produced the best recovery result of the cubic and quarter-cubic pattern with a grade of 2 at 75 °C; and worst result was from the line pattern, with a grade of 7 at 65 °C. In terms of time, the cross, cubic-subdivision, grid and gyroid pattern took the shortest duration of 12 s at 75 °C; and the concentric pattern took the longest duration of 28 s at 65 °C. Next, the experiments showed that a 80% infill density produced the best recovery result of the grid and quarter-cubic pattern with a grade of 1 at 75 °C; and worst result of the cubic-subdivision and line pattern, with a grade of 7 at 65 °C. In terms of time, the cross pattern took the shortest duration of 12 s at 75 °C; and the concentric pattern took the longest duration of 29 s at 65 °C. Lastly, the experiments showed that a 100% infill density produced the best recovery result of the octet and quarter-cubic pattern, with a grade of 1 at 70 °C and 75 °C; and worst result was the cubic-subdivision pattern, with a grade of 8 at 65 °C. In terms of time, the cross and cubic-subdivision pattern took the shortest duration of 13 s at 75 °C; and the concentric pattern took the longest duration of 29 s at 65 °C.

The experiments showed that the best recovery result came from an infill density of 100% for the octet and quarter-cubic pattern with a grade of 1; and the worst recovery result came from an infill density of 20% for the line pattern, with a grade of 9. The shortest period of recovery time was achieved from an infill density of 20% for the cubic-subdivision pattern with a time of 6 s; and longest period of recovery time was achieved from an infill density of 100% for the concentric pattern, with a time of 29 s. The findings are in line with Yang et al. [21], who investigated the relationship between the part density and extrusion temperature, concluding that dense SMP structures caused greater recovery stress during recovery.

**Figure 8:**
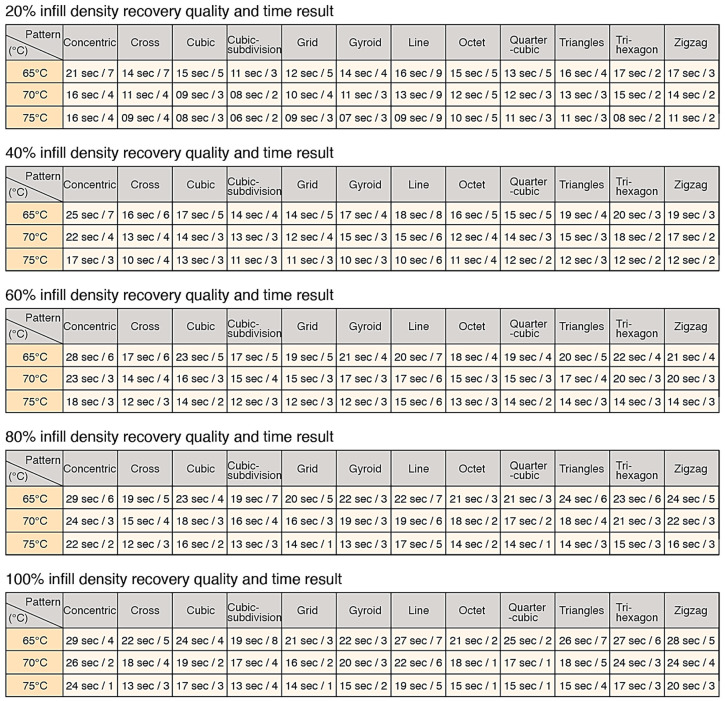
Results from 5 infill density showing recovery quality and time.

To disseminate the findings, and to make the results usable by the research community, the collected information was compiled in the form of a matrix, for designers, engineers and manufacturers to be able to appreciate and implement the possibilities of applying shape changing behaviors when developing 4DP parts. This is achieved by the selection of the material, the print pattern and the infill density, as well as the activation temperature. In addition, the information gathered would help users to determine whether the accuracy of the shape recovery or the time it took to return to its original form was more important. This toolkit has two main advantages. Firstly, 4DP parts can be designed and programmed through the control and selection of patterns and infill densities. Secondly, it can potentially facilitate the diagnosis of errors in the print output, and to analyze the interaction between the programming elements and the AM machine. The initial information from the experimental results described in Section 2.4 was quantified in the table shown in Figure 8.

**Figure 9:**
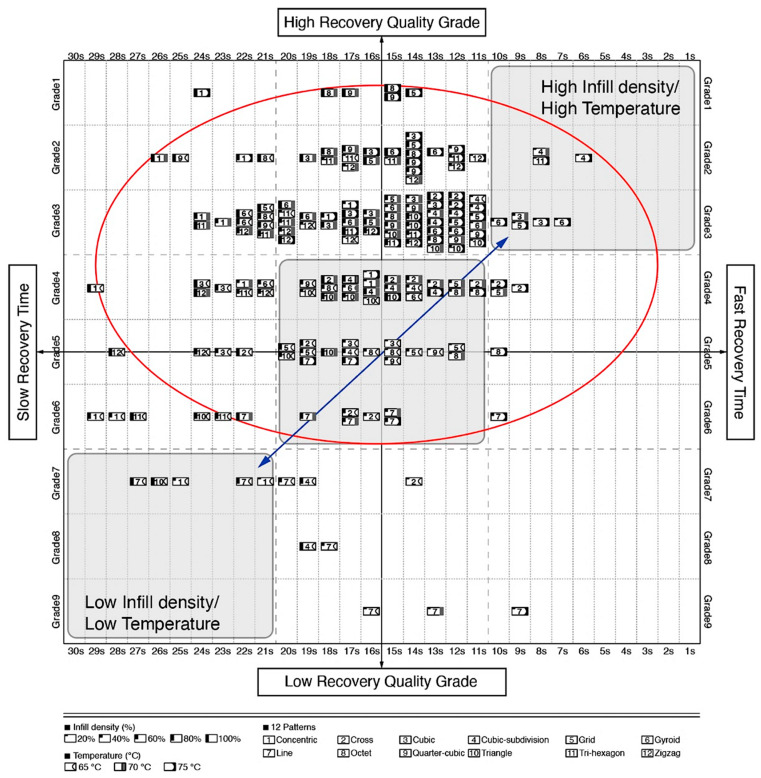
Shape recovery result and time distribution according to printing parameters.

It was then represented as a graph in Figure 9 as a single visual reference guide. As shown in the bottom of Figure 9, the distribution diagram from the results was divided according to the infill density, temperature and pattern. The 12 patterns were expressed in the form of a numerical value from 1 to 12. Patterns according to numbers are arranged alphabetically. No. 1—Concentric pattern, No. 2—Cross Line pattern, No. 3—Cubic pattern, No. 4—Cubic-subdivision pattern, No. 5—Grid pattern, No. 6—Gyroid pattern, No. 7—Line pattern, No. 8—Octet pattern, No. 9—Quarter-cubic pattern, No. 10—Triangle pattern, No. 11—Tri-hexagon pattern and No. 12—Zigzag pattern. The *X*-axis represents the time it takes for the part to recover to its original printed shape. The *Y*-axis represents the grade of the shape recovery result, and the lower the grade, the better the shape recovery. The graph shows the distribution of a total of 180 shape recovery, and the time results with 5 print densities, 12 print patterns and 3 shape recovery temperatures of 65 °C, 70 °C and 75 °C. The results with excellent shape recovery and fast recovery time are shown in the upper right of the matrix. Conversely, the results of poor shape recovery and slow recovery time are shown at the bottom left of the matrix. The blue arrows indicate the sequential direction of the printing parameters. As a result of the printing parameters, the shape recovery result and time are generally evenly distributed in the graph, as shown within the red circle. This means that except for a specific pattern, the print parameters greatly affect the shape recovery quality result and time.

**Figure 10:**
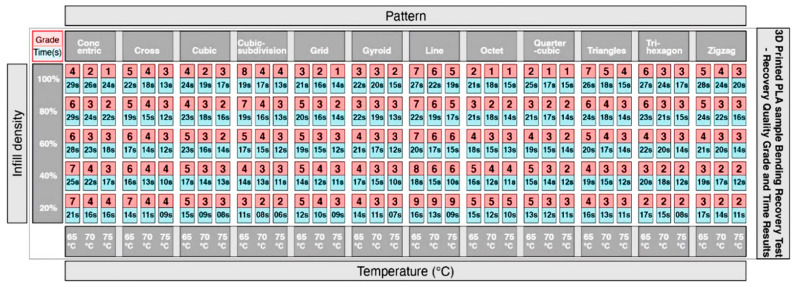
Graphical table representing the results of 12 patterns and 5 infill densities.

The information from Figure 9 was further developed into a graphical layout in Figure 10, to improve access to the information. The single digits in the horizontal rows colored in red describe the grade of the shape recovery result, where a lower grade represents better shape recovery. The horizontal rows colored in blue describe the time taken for the parts to recover to its original printed shape. Elements of the 12 print patterns, as well as the 5 print densities, are also illustrated in the image, in addition to 3 different shape recovery temperatures, of 65 °C, 70 °C and 75 °C. Figure 10 shows that the lowest recovery grade pattern in the 20% infill density is the line pattern (since it has a numerical value of 9), with poor results at 65 °C, 70 °C and 75 °C. Similarly, the longest recovery time was recorded by observing the concentric pattern with a 100% infill density, taking over 65 s and at 65 °C. Using this graphical method, it is possible to check all the recorded results from the 5 infill densities, 12 patterns, and 3 different temperatures.

**Figure 11:**
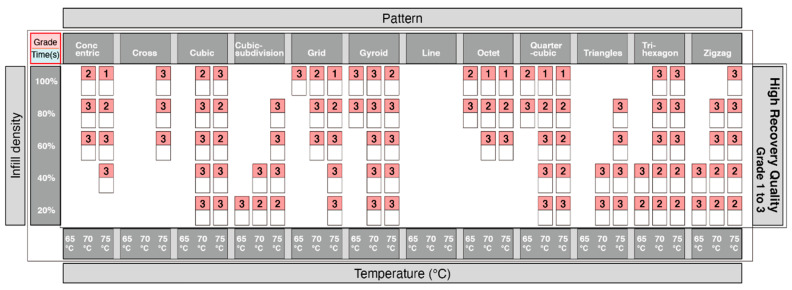
Graphical table representing best results of shape recovery.

**Figure 12:**
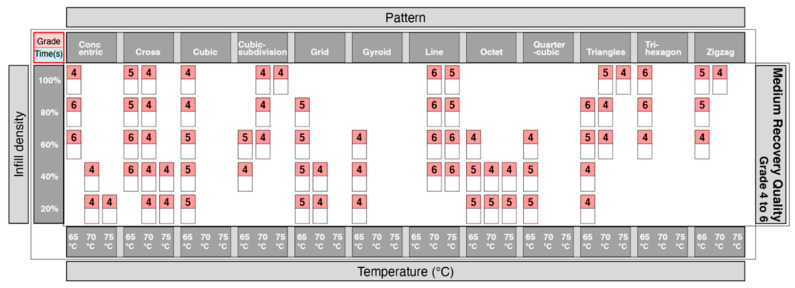
Graphical table representing moderate results of shape recovery.

**Figure 13:**
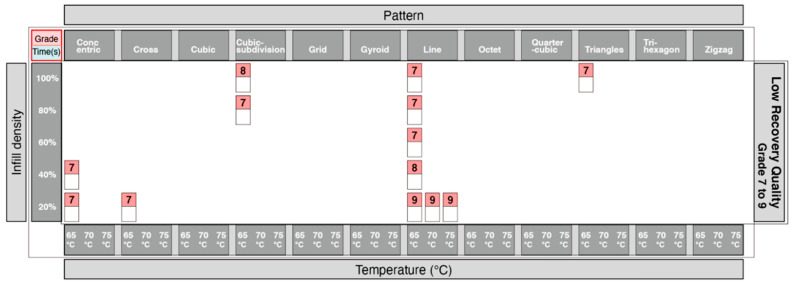
Graphical table representing poor results of shape recovery.

From the table in Figure 10, the quarter-cubic and octet print patterns showed the best recovery quality, and the line pattern showed the worst recovery quality. Taking it a step further, we also wanted to analyze which print patterns could provide a good recovery of 4DP parts. The information showing the quality of recovery was divided into 3 groups. Grades from 1–3 (Figure 11) showed the highest quality of recovery and reflected with the represented print patterns. Grades from 4–6 (Figure 12) showed a medium quality of recovery; and grades from 7–9 (Figure 13) showed print patterns that represent the worst quality of recovery.

**Figure 14:**
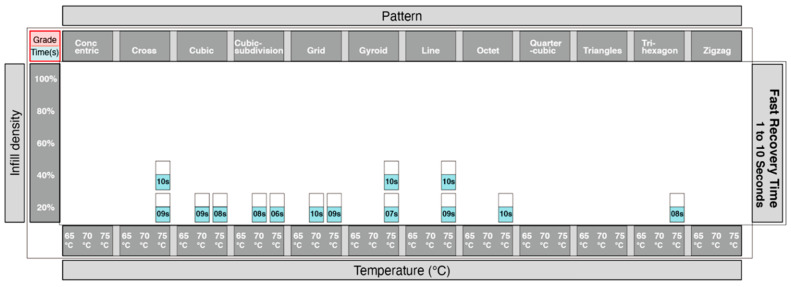
Graphical table representing the shortest recovery time.

**Figure 15:**
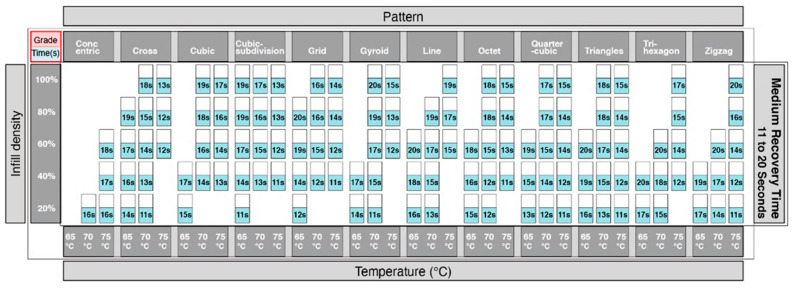
Graphical table representing moderate recovery time.

**Figure 16:**
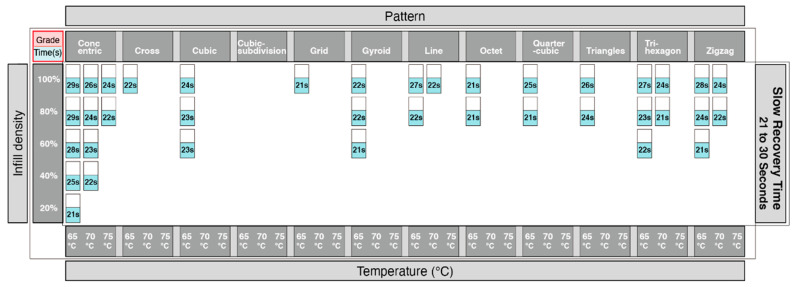
Graphical table representing the longest recovery time.

Adopting the same approach using the data from Figure 10, we were able to identify which print patterns influenced the recovery time of the 4DP parts. The information showing the time taken for recovery was divided into 3 groups. Figure 14 showed the shortest time for recovery, Figure 15 with a moderate time for recovery, and Figure 16 with the longest time for recovery. Moreover, 1–10 s was the fastest recovery time, 11–20 s reflects a moderate recovery time, and 21–30 s reflects the longest recovery time. The shortest recovery time is mainly due to a low percentage of infill density of 20% and 40%; and using high temperatures with patterns such as cross, cubic-subdivision, grid, gyroid, line, octet and tri-hexagon being used. The moderate recovery time took up the largest proportion. Slow recovery times were mainly due to having a high percentage of infill density of 80% and 100% and with a low temperature. Therefore, a low percentage infill density with a high temperature results in a much shorter recovery time. Conversely, a high percentage infill density with a low temperature would result in a slow recovery time. In conclusion, the results show that the recovery time is largely influenced by the pattern, infill density and the temperature.

**Figure 17:**
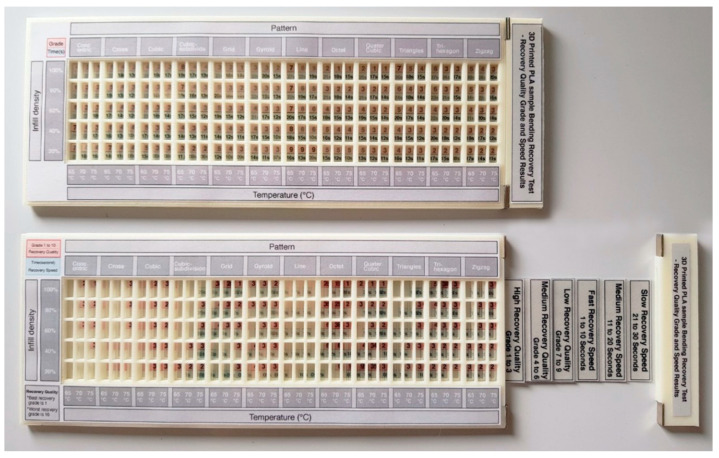
Physical version of the 4D Printing toolkit.

Finally, a physical version of the toolkit in Figure 17 was produced, with each of the information sheets from Figure 10, Figure 11, Figure 12, Figure 13, Figure 14, Figure 15 and Figure 16 printed onto transparent acetate film, so that users could intuitively filter information or identify overlapping areas. This physical version was tested with potential users for feedback, which received a very positive response for the accuracy of information and ease of use. However, future work would aim to undertake more thorough testing of the kit, involving more participants, and with a view of making this more widely accessible and available in a digital format.

## 4. Discussion

**Figure 18:**
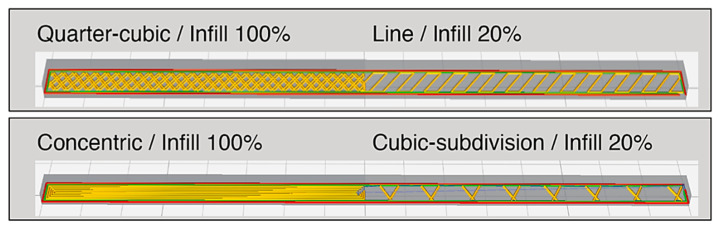
A computer generated file showing a combination of an assumed best and worst recovery setting (**top**), as well as fastest and slowest recovery time (**bottom**).

In conclusion, the print pattern and infill density of PLA samples were tested using the same water-bath process to ensure consistency and rigor. The purpose of the test was to confirm the findings from Section 3, and to also validate the use of the toolkit. Due to time and resource constraints, it was not possible to evaluate every single element. An objective comparison method was used, in which the pattern and infill density of the highest and lowest recovery results, and the pattern and infill density of the fastest and slowest recovery time, would be tested. The ‘Quarter-cubic’ pattern with a 100% infill density (with an assumed best recovery result); and the ‘Line’ pattern with a 20% infill density (with an assumed worst recovery result) were selected. In addition, the ‘Cubic-subdivision’ pattern with a 20% infill density (with an assumed shortest recovery time); and the ‘Concentric’ pattern with a 100% infill density (with an assumed longest recovery time) were selected (Figure 18). The PLA samples were fabricated using the same Qidi X-Pro printer using the identical print parameters. To further extend our understanding of the subject matter, and to validate the results, each sample would contain two different print patterns with two different infill densities using a rectangular sample of 160 mm × 6 mm × 3 mm (each pattern consists of 80 mm × 6 mm × 3 mm). Having two different patterns and infill densities in a single sample should show a clear difference when they are immersed in the water bath.

**Figure 19:**
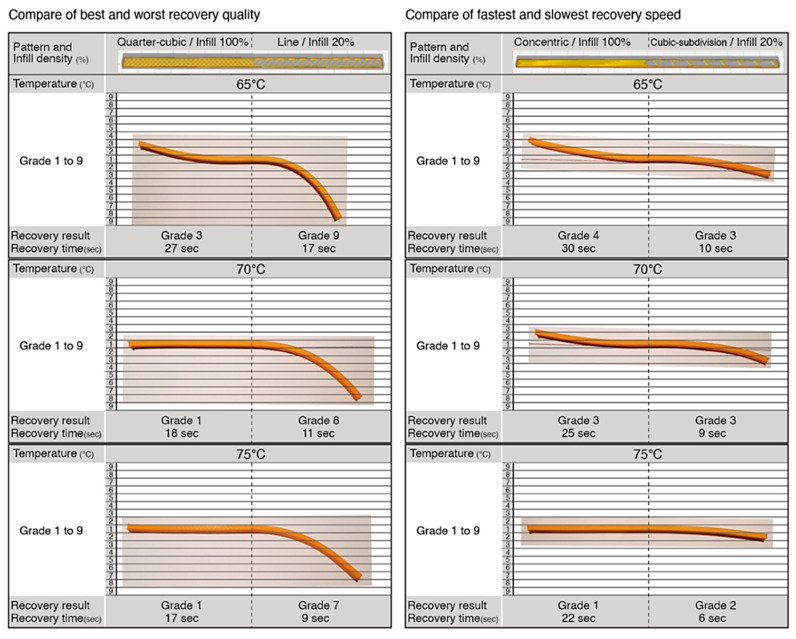
Physical samples showing the combination of mixed pattern and different infill densities.

After the physical samples were produced, the results were similar from the previous experiments. In terms of recovery quality, the ‘Line’ pattern achieved a grade of 9 at 65 °C, a grade of 8 at 70 °C, and a grade of 7 at 75 °C, showing a slight difference of grades 1 and 2 from the previous experiment. The ‘Quarter-cubic’ pattern recovery quality showed the same results, of a grade of 3 at 65 °C, a grade of 1 at 70 °C, and a grade of 1 at 75 °C. In terms of recovery time, there was only a difference of between one to two seconds when compared to earlier experiments. The comparison of the ‘Concentric’ pattern and the ‘Cubic-subdivision’ pattern also showed similar results. The ‘Concentric’ pattern achieved a grade of 4 at 65 °C, a grade of 2 at 70 °C, and a grade of 1 at 75 °C. The ‘Cubic-subdivision’ pattern achieved a grade of 3 at 65 °C, a grade of 2 at 70 °C, and of grade 2 at 75 °C. Both patterns showed the same recovery quality, except 70 °C, as shown in Figure 19. In terms of recovery time, there was only a difference of one to two seconds when compared to earlier experiments. The samples on the left in Figure 19 show the use of patterns that display the best and worst recovery results; and the samples on the right display the fastest and slowest recovery time. The results are in line with Wu et al [16], who conducted an experiment on the influence of process parameters of the SME. The results revealed that the SME of AM parts using PLA were mostly influenced by the recovery temperature, followed by the deformation temperature and print parameters (layer thickness and raster angle). However, results from this research showed that parameters such as pattern and infill density also largely affect the SME. Further experiments are still required to verify the results with greater accuracy and repeatability.

## 5. Conclusions

**Figure 20:**
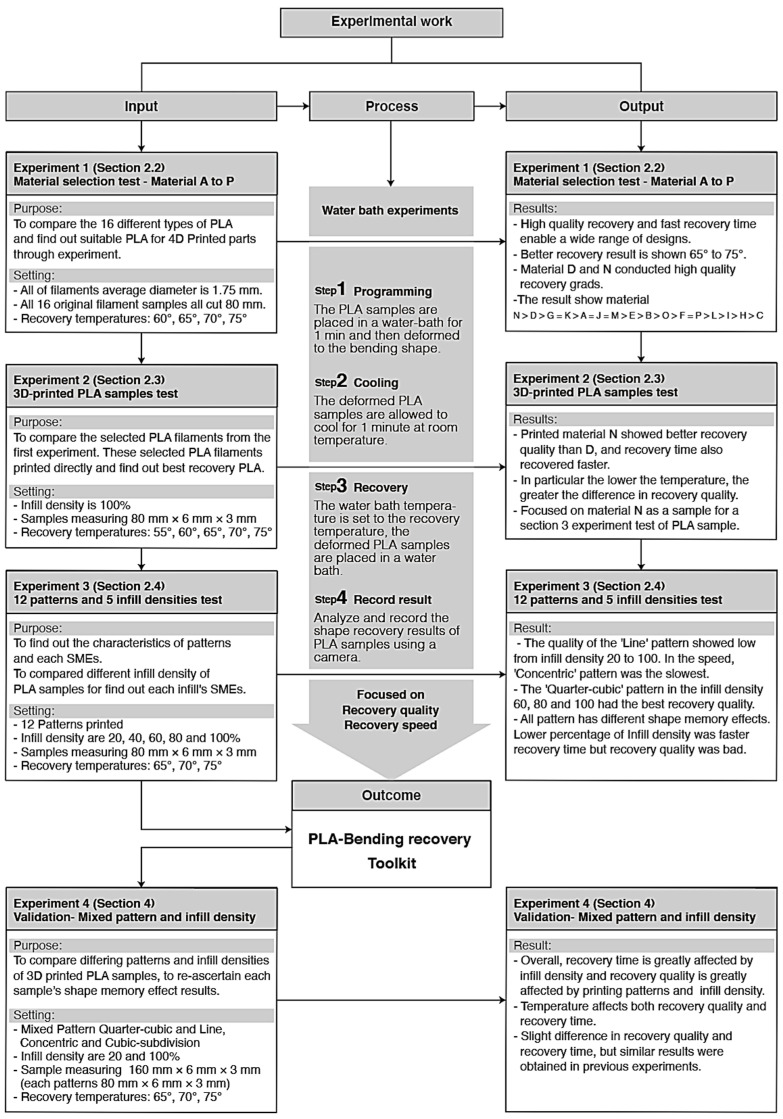
The flowchart showing the inputs, processes, outputs and outcomes.

This research has enabled a statistical approach of categorizing the SME of 16 different filaments, followed by tailoring the SME through the print pattern and infill density. The findings provide designers, engineers and manufacturers with a fundamental understanding that print parameters of pattern and infill density significantly influence the shape recovery quality and the recovery time of 4DP parts. This experiment shows that the use of different infill patterns has a different resultant SME. The temperature affects both recovery quality and recovery time. A low percentage infill density with a high temperature results in a much shorter recovery time. Conversely, a high percentage infill density with the low temperature would result in a slow recovery time. In general, a high temperature and high infill density provide better recovery quality, and a low temperature and low infill density result in low recovery quality. In conclusion, Figure 20 presents a flowchart that documents the entire process of this research. At the input stage, four experiments were conducted sequentially, and each result was performed using the same water bath. Although these experiments have provided a fundamental basis that the print parameters of pattern and infill density influence the shape recovery quality and the recovery time, there are still limitations of this study. For example, even more trials involving different PLA materials, the size and shape of the sample, and other types of shape changing behaviors and stimuli still need to be investigated. This study has provided an understanding of the SME in the use of 4DP parts, to enable better control of the shape change behavior through intelligent design and the modeling of CAD.
